# Marjolin’s ulcer: a preventable malignancy arising from scars

**DOI:** 10.1186/1477-7819-11-313

**Published:** 2013-12-17

**Authors:** Nanze Yu, Xiao Long, Jorge R Lujan-Hernandez, Kazi Z Hassan, Ming Bai, Yang Wang, Xiaojun Wang, Ru Zhao

**Affiliations:** 1 Division of Plastic Surgery, Peking Union Medical College Hospital, Peking Union Medical College and Chinese Academy of Medical Science, No.1 Shuaifuyuan, Dongcheng District, Beijing 100730, China; 2 Division of Plastic Surgery, Brigham and Women’s Hospital and Harvard Medical School, 75 Francis Street, Boston MA 02115, USA

**Keywords:** Marjolin’s ulcer, Squamous cell carcinoma

## Abstract

**Background:**

Marjolin’s ulcer (MU) is a rare malignancy arising from various forms of scars. This potentially fatal complication typically occurs after a certain latency period. This article attempts to reveal the importance of the latency period in the prevention and early treatment of the malignancy.

**Methods:**

A retrospective review of 17 MU patients who underwent surgical procedures between June of 2005 and December 2011 was conducted. Etiology of injuries, latency period, repeated ulceration, and outcomes were recorded. This observational report reveals characteristics of patients who develop MU.

**Results:**

An incidence of 0.7% of MU was found amongst patients complaining of existing scars in our study; burns and trauma were the most common etiology of MU. The mean latency period was 29 years (SD = 19) and the mean post-ulceration period was 7 years (SD = 9). Statistical analysis revealed a negative correlation between the age of patients at injury and the length of latency period (r = −0.8*, P* <0.01), as well as the lengths of pre-ulceration and post-ulceration periods (r = −0.7, *P* <0.01).

**Conclusions:**

Patients experience different lengths of pre- and post-ulceration periods during the latency period. Younger patients tend to have a longer latency period. Skin breakdown on chronic scars and chronic unhealed ulcers are two main sources of MU. MU may be preventable with a close surveillance of the ulcer during the latency period.

## Background

Marjolin’s ulcer (MU) refers to a rare but highly aggressive ulcerating squamous cell carcinoma (SCC) that is most often presented in an area of chronic burn wounds [1]. However, it is also associated with chronic inflammatory states, as in non-healing wounds, venous ulcers, lupus vulgaris, vaccination scars, snake bite scars, pressure sores, osteomyelitis zones, pilonidal abscess, and radiotherapy areas [2-4]. Celsus first described the malignant transformation of the thermal burn scar from his observations dating back to the 1^st^ century A.D. [5]. In the 19^th^ century, the French surgeon Jean-Nicholas Marjolin demonstrated the cellular changes of ulcerated lesions in scar tissue [6], but it was not until Robert Smith in 1850 that a detailed description of the pathology was published and named “Marjolin’s ulcer” [7,8].

**Figure 1:**
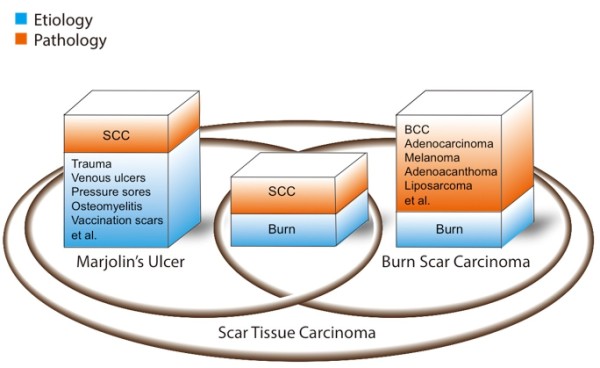
**Comparison of the concepts of MU, burn scar carcinoma, and scar tissue carcinoma.** The intersection between MU and burn scar carcinoma represents the etiology and pathological outcome in common. SCC = squamous cell carcinoma; BCC = basal cell carcinoma.

Although it has been more than 160 years since the eponym was first used, the disagreement on the use of the term “Marjolin’s ulcer” synonymously with burn scar carcinoma still exists [9]. The classic definition of MU only applies to the squamous cell variant [1,10,11], whereas the term “scar tissue carcinoma” is used for all malignancies arising in scars (Figure 1) [12]. Although some authors hold that MU may also refer to the latter in a broad sense [13-16], the use of this term as a name for different clinical entities may not be appropriate. As numerous skin carcinomas have been found, their onset, signs, clinical features, progression, and treatments can be completely different.

**Figure 2:**
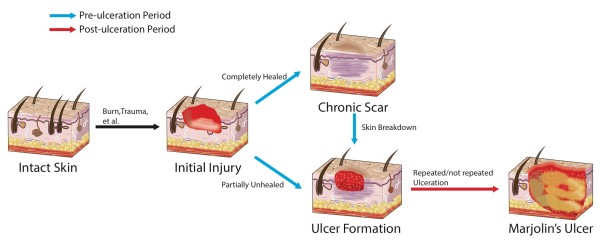
**Various paths to malignancy during the latency period.** Ulcer formation is a key step between initial injury and final malignant transformation.

During the course of MU, the latency period is defined as the time between initial injury and the confirmation of a pathologic diagnosis of malignancy [1]. In our study, we divided the latency period into two parts: a “pre-ulceration period” referring to the period from initial injury to the appearance of an ulcerated lesion, and “post-ulceration period” referring to the period from ulceration to the diagnosis of SCC by a positive biopsy (Figure 2). By analyzing data of 17 cases, we aimed to reveal the role of the latency period in the prevention and early detection and treatment of MU.

## Methods

In the period from June of 2005 and December 2011, 2,984 patients with scars of different causes and no previous history of cancer were seen in the plastic surgery division, at Peking Union Medical College Hospital, China. All scars, regardless of appearance, underwent biopsy/pathological examination leading to the diagnosis of 17 cases of MU. The majority of patients that were diagnosed complained of chronic, disfiguring, non-healing ulcers. Our therapeutic regimes were planned according to available guidelines, clinical evaluation, biopsy results, surgical expertise, and radiologic findings in a multidisciplinary approach with other departments from our hospital.

Ten male and seven female patients ranging from 34 to 87 years old were diagnosed with MU. Characteristics and areas of ulcers were recorded. These patients were followed-up for an average of 2.9 years (1 to 6 years) after treatment. Their medical records were reviewed and stratified retrospectively. Demographic and relevant clinical information is presented in Table 1, including etiology, the latency period, pre- and post-ulceration periods, and outcome.

**Table 1:** Demographic characteristics of all the 17 MU cases

**Patient**	**Age (years)**	**Gender**	**Etiology**	**Latency period (years)**	**Pre-ulceration period (years)**	**Cause for ulceration**	**Repeated or not**	**Localization**	**Lesion appearance**	**Treatment**	**Outcome**
1	56	M	Flame burn	27	0	Incomplete healing	Yes	Left leg	Ulcerated	ALT flap	Tumor-free for 5 years
2	87	M	Venous ulcer	30	0	Spontaneous	Yes	Right leg	Ulcerated	Skin grafting	Tumor-free for 2 years
3	71	M	Trauma	10	0	Incomplete healing	Yes	Right gluteal	Ulcerated	SGAP flap	Death
4	70	F	Oil burn	1	0	Incomplete healing	Yes	Face	Ulcerated	Local flap	Tumor-free for 3 years
5	82	M	Trauma	2	0	Incomplete healing	Yes	Right hand	Ulcerated	Skin grafting	Tumor-free for 6 years
6	60	F	Trauma	8	0	Incomplete healing	Yes	Gluteal	Ulcerated	Skin grafting	Death
7	71	M	Trauma	10	5	Spontaneous	Yes	Left hand	Ulcerated	Skin grafting	Tumor-free for 2 years
8	59	F	Infection	30	10	Spontaneous	Yes	Back	Ulcerated	Skin grafting	Lost to follow-up
9	34	M	Flame burn	32	13	Spontaneous	Yes	Scalp	Ulcerated	Skin grafting	Death
10	42	M	Steam burn	15	15	Spontaneous	No	Right leg	Ulcerated	Skin grafting	Lost to follow-up
11	35	F	Flame burn	35	29	Accidental abrasion	Yes	Scalp	Ulcerated	ALT flap	Tumor-free for 1 year
12	57	F	Trauma	42	41	Accidental abrasion	No	Left hand	Ulcerated	Skin grafting	Recurrence 3 years later
13	48	F	Infection	42	42	Scratching	No	Scalp	Ulcerated	Skin grafting	Lost to follow-up
14	70	M	Steam burn	45	45	Spontaneous	No	Right leg	Ulcerated	Skin grafting	Tumor-free for 4 years
15	78	M	Flame burn	45	45	No	No	Chest	Exophytic	Skin grafting	Death
16	68	M	Infection	58	58	Spontaneous	No	Gluteal	Ulcerated	SGAP flap	Recurrence 1 year later
17	62	F	Flame burn	61	60	Spontaneous	No	Scalp	Ulcerated	Skin grafting	Tumor-free for 2 years

ALT flap = anterolateral thigh flap; SGAP flap = superior gluteal artery perforator flap.

All statistical algorithms were developed using the R Project for statistical computing. Two-tailed Spearman correlation was used, and *P* <0.05 was considered statistically significant.

The retrospective study was approved by the Institutional Review Board of Peking Union Medical College Hospital. Written informed consent was obtained from each patient involved in this study.

## Results

### Patient history

Marjolin’s ulcer was diagnosed in patients with a mean age of 62 years (34–87, SD = 15). In 82% of cases, more than 10 years elapsed during the latency period, with an average time of 29 years (SD = 19). However, an acute form of presentation was seen in one patient (6%) diagnosed 1 year after the initial injury. The mean pre-ulceration period was 21 years (SD = 22). The mean post-ulceration period was 8 years (SD = 10).

**Figure 3:**
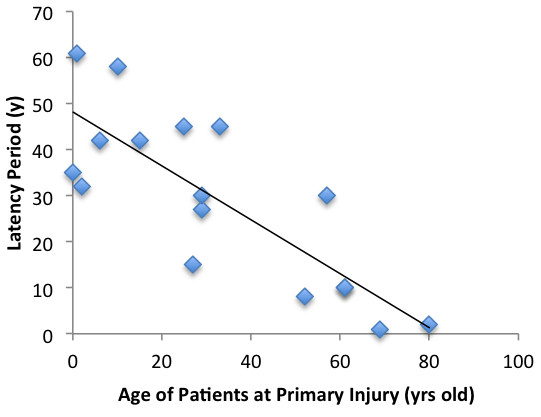
Relationship between the age of patients at initial injury and the length of the latency period.

**Figure 4:**
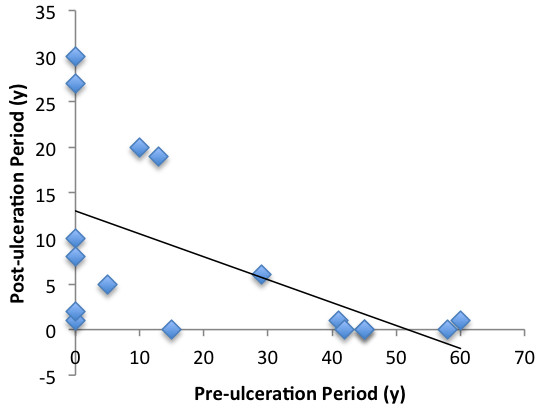
Relationship between the pre- and post-ulceration periods.

Statistical analysis revealed a negative correlation (r = −0.8) between the age of patients at which the initial injury occurred and the length of the latency period (*P* <0.01) (Figure 3). Similarly, a negative correlation (r = −0.7) was found between the pre- and post-ulceration periods (*P* <0.01) (Figure 4).

The main etiology was post-burn scars, which resulted from flame (29%), steam (12%), and oil (6%) injuries. The other diverse sources were trauma (29%), skin infections (18%), and venous ulcers (6%). The most affected sites were the scalp (24%), lower limbs (24%), upper limbs (18%), and gluteal region (18%). Infection was reported in seven patients (41%) and *Staphylococcus aureus* was isolated in cultures.

### Treatment

**Figure 5:**
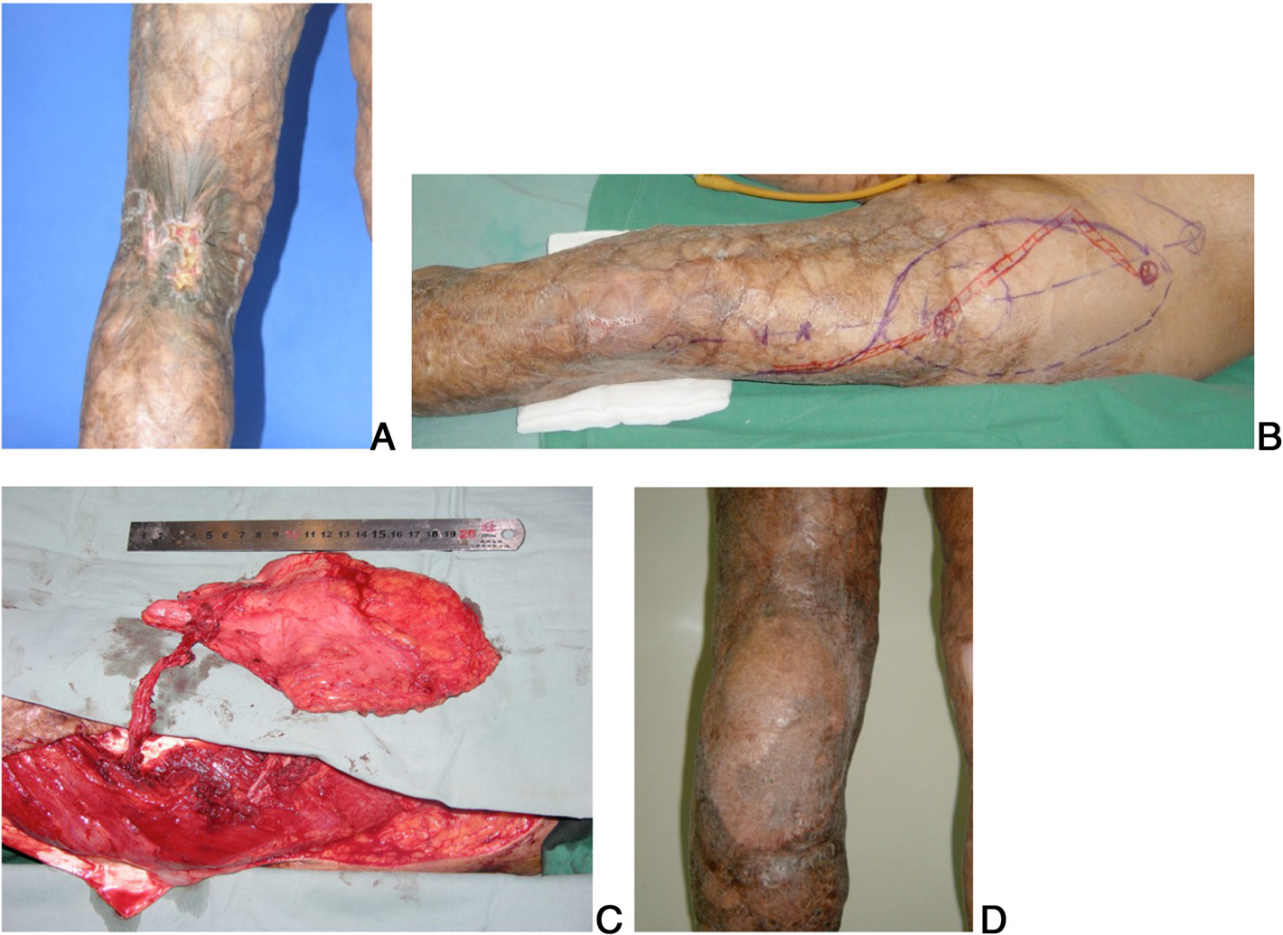
**Anterolateral thigh (ALT) flap reconstruction. (A)** A 56-year-old male MU patient burned his left leg at the age of 29. **(B)** Preoperative design of a reverse-flow ALT flap. **(C)** Reverse-flow ALT flap measured 20 cm × 10 cm, was elevated during operation. **(D)** Area of ulceration widely excised and reconstructed with ALT flap three years postoperatively.

**Figure 6:**
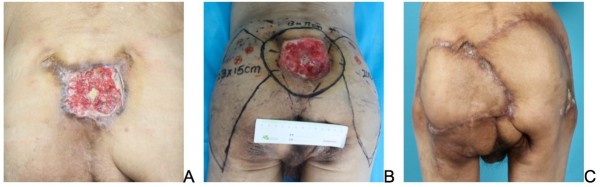
**Superior gluteal artery perforator (SGAP) flap reconstruction. (A)** A 68-year-old male gluteal MU patient injured at the age of 58. **(B)** Preoperative design of bilateral inferior gluteal artery perforator flaps. **(C)** Area of ulceration widely excised and covered with good results.

**Figure 7:**
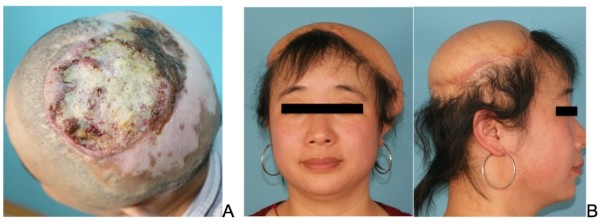
**Anterolateral thigh (ALT) flap reconstruction. (A)** A 35-year-old female MU patient had her scalp burned at the age of 6. **(B)** Anterior and lateral views of the patient, one year after wide excision and reconstruction with ALT flap.

Surgical treatment was given to all patients. Wide excision with 3 cm of free margins to the border/base of the ulcer was performed in every case. Reconstruction was performed with split-thickness skin grafts (71%) or musculocutaneous/cutaneous flaps (29%) depending on the wound condition after excision (Figures 5, 6, and 7). Excision of the external lamina was carried out for one patient with skull invasion. Radiotherapy was applied for advanced cases with ulcers of a diameter >10 cm, as suggested by the oncology-radiotherapy department. Regional lymph nodes were all carefully assessed by palpation and superficial inguinal lymph node dissection was performed in one clinically suspicious case. The pathological result was negative.

### Follow-up/recurrence

During follow-up, three patients were lost to follow-up and eight cases remained tumor-free. Four cases died within one year after operation due to recurrence and metastasis. Local recurrence was found in two cases; one had MU on the left hand, and experienced another ulceration of SCC on the left foot. The other presented a new ulcerated mass, 4 cm away from the previous grafted region. Both patients were re-admitted and treated with wide re-excision and skin grafting.

## Discussion

MU is a rare and aggressive cutaneous malignant transformation with an incidence of 0.1% to 2.5% after a long-term inflammatory or traumatic insult to the skin [16,17]. The main etiology tends to be post-burn scars and traumatic wounds [1]. Even though several theories have been postulated to elucidate the mechanisms generating this process, none can fully explain it [18]. Nevertheless, there is a consensus on the importance of chronic irritation. Repeated ulceration to the scar and subsequent initiation of re-epithelialization provides a prolonged stimulus for cellular proliferation and may increase the rate of spontaneous mutations [17,19,20].

An association between latency period and malignant transformation was first suggested by Lawrence [21]. Along our practice, we realized that a typical latency period of MU could be divided into two parts: pre- and post-ulceration periods, both of which have been considered to correlate in some way during the course of MU. Patient observations have also supported that the length of the pre- and post-ulceration periods may vary from one case to another.

After a certain period of existence of a chronic scar, early stages of MU usually present with symptoms of burning and itching, followed by blisters and prurigo. During this period, which we called the “pre-ulceration period”, the surface of the scars remains intact. The duration of the pre-ulceration period, or “the age of the scar”, may be important for the prognosis of MU [22].

A new ulcer forms whenever the integrity of skin is compromised by spontaneous rupture, scratching, or lack of self-care. After ulceration, some patients will experience repeated cycles of healing followed by rupturing of skin, which is called repeated ulceration period [7]. At this stage, ulcers protrude and deepen, accompanied with severe pain, purulent discharge, foul odor, and bleeding. Malignant transformation of chronic ulcers is closely related to the duration of ulceration. This means that the longer the ulcer duration, the higher the risk of dysplasia [23]. Moreover, young patients tend to have a course of cycling between skin breakdown and repair before developing malignancy [21].

In our study, six patients (35%) reported that a small portion of their initial injury never completely healed, which means they experienced repeated ulceration shortly after the initial injury. Therefore, the length of the post-ulceration period was almost as long as that of the latency period. While SCC can arise from these kinds of unhealed chronic ulcers, it is not an uncommon source for MU [9,24].

We found that the shorter the pre-ulceration period, the longer the post-ulceration period will be, and vice versa. Moreover, a chronic scar has a higher likelihood of developing carcinoma at the first ulceration. Prolonged healing of the primary injury, that is, a prolonged post-ulceration period is also a major potential risk factor for the development of MU. Since biopsy remains the gold standard for the diagnosis of MU [25,26], it should be applied for suspicious lesions that have not healed in 3 months.

A customized treatment regime should be designed after carefully considering the clinical evaluation, pathologic, and imaging results [27]. MU is more aggressive than primary skin tumors, therefore nodal assessment and wide surgical excision are recommended [28]. As recurrence of MU is higher than normal SCC (11–37%), 2–4 cm of free margins should be resected [2,3,7,29]. Frozen biopsy during the operation is helpful, for MU always occurs in varying depth [22,30]. Lifelong follow-ups should be conducted according to our experience.

Moreover, since the majority of MUs occur in long-duration unstable scars of ungrafted full-thickness burns [31], the joint regions, especially flexion creases, are more commonly involved due to predisposition to activity-related repeated ulceration [1,7,32]. Early surgical management could also achieve a possible prevention strategy.

## Conclusions

MU is a rare but highly aggressive ulcerating SCC. The formation of an ulcer in the latency period plays an important role in the course of MU. Based on the different length of the pre- and post-ulceration periods during the latency period, skin breakdown on chronic scars and chronic unhealed ulcers are two main sources of MU. This potentially fatal complication may be preventable and treatable by surgical management of initial injuries and early diagnosis and treatment of unhealed ulcers. Patients should be followed-up for the rest of their life, as MU is more aggressive than initial skin carcinomas.

## Abbreviations

ALT flap: Anterolateral thigh flap; BCC: Basal cell carcinoma; SGAP flap: Superior gluteal artery perforator flap; MU: Marjolin’s ulcer; SCC: Squamous cell carcinoma.

## Competing interests

The authors declare that they have no competing interests.

## Authors’ contributions

NY: Conception and design, collection and assembly of data, data analysis and interpretation, manuscript writing. XL: Conception and design, manuscript writing. JRL-H: Data analysis and interpretation, manuscript writing. KZH: Data analysis and interpretation, manuscript writing. MB: Design, manuscript writing. YW: Collection of data. XW: Conception and design, data analysis and interpretation, manuscript writing. RZ: Conception and design, financial support, data analysis and interpretation, manuscript writing, final approval of manuscript. All authors read and approved the final manuscript.

## References

[B1] Kerr-Valentic MA Samimi K Rohlen BH Agarwal JP Rockwell WB Marjolin’s ulcer: modern analysis of an ancient problem Plast Reconstr Surg 2009 11 184 191 10.1097/PRS.0b013e3181904d86 19116552

[B2] Bozkurt M Kapi E Kuvat SV Ozekinci S Current concepts in the management of Marjolin’s ulcers: outcomes from a standardized treatment protocol in 16 cases J Burn Care Res 2010 11 776 780 10.1097/BCR.0b013e3181eed210 20661151

[B3] Kadir AR Burn scar neoplasm Ann Burns Fire Disasters 2007 11 185 188 21991095 PMC3188084

[B4] Copcu E Aktas A Sisman N Oztan Y Thirty-one cases of Marjolin’s ulcer Clin Exp Derm 2003 11 138 141 10.1046/j.1365-2230.2003.01210.x 12653697

[B5] Tio J Clarkson JHW Mirsa A Srivastava S Malignant change after 18 months in a lower limb ulcer Br J Plast Surg 2003 11 825 828 10.1016/j.bjps.2003.08.016 14615262

[B6] Marjolin JN Ulcere Dictionnaire de Medicine. Vol. 21 1828 Paris: Becher 31 50

[B7] Huang CY Feng CH Hsiao YC Chuang SS Yang JY Burn scar carcinoma J Dermatolog Treat 2010 11 350 356 10.3109/09546630903386580 20438387

[B8] Smith RW Observations upon the warty ulcer of Marjolin Dublin Quart J Med Sci 1850 11 257 275

[B9] Kowal-Vern A Criswell BK Burn scar neoplasms: a literature review and statistical analysis Burns 2005 11 4 403 413 10.1016/j.burns.2005.02.015 15896501

[B10] MacNalty SAS British Medical Dictionary 1961 London: Butterworth 876

[B11] Cobey FC Engrav LH Klein MB Isom CN Byrd DR Brief report: sentinel lymph node dissection and burn scar carcinoma sentinel node and burn scar carcinoma Burns 2008 11 2 271 274 10.1016/j.burns.2006.09.006 17374455

[B12] Arons MS Lynch JB Lewis SR Blocker TG Jr Scar tissue carcinoma. I. A clinical study with special reference to burn scar carcinoma Ann Surg 1965 11 170 188 10.1097/00000658-196502000-00003 14260013 PMC1408939

[B13] Phillips TJ Salman SM Burn scar carcinoma Dermatol Surg 1998 11 561 565 9598012

[B14] Turegun M Nisanci M Guler M Burn scar carcinoma with longer lag period arising in previously grafted area Burns 1997 11 496 497 10.1016/S0305-4179(97)00041-7 9429029

[B15] Baldursson BT Hedblad MA Beitner H Lindelöf B Squamous cell carcinoma complicating chronic venous leg ulceration: a study of the histopathology, course and survival in 25 patients Br J Dermatol 1999 11 1148 1152 10.1046/j.1365-2133.1999.02879.x 10354087

[B16] Dupree MT Boyer JD Cobb MW SC arising in a burn scar Cutis 1998 11 49 51 9675536

[B17] Gül U Kiliç A Squamous cell carcinoma developing on burn scar Ann Plast Surg 2006 11 406 408 10.1097/01.sap.0000200734.74303.d5 16557073

[B18] Nthumba PM Marjolin’s ulcers: theories, prognostic factors and their peculiarities in spina bifida patients World J Surg Oncol 2010 11 108 10.1186/1477-7819-8-108 21129225 PMC3014936

[B19] Fairbairn Hill BB Sloan DA Lee EY McGrath PC Kenady DE Marjolin’s ulcer of the foot caused by nonburn trauma South Med J 1996 11 7 707 710 10.1097/00007611-199607000-00011 8685758

[B20] Fairbairn NG Hamilton SA Management of Marjolin’s ulcer in a chronic pressure sore secondary to paraplegia: a radical surgical solution Int Wound J 2011 11 5 533 536 10.1111/j.1742-481X.2011.00831.x 21827630 PMC7950692

[B21] Lawrence EA Carcinoma arising in the scars of thermal burns Surg Gynecol Obstet 1952 11 579 588 12995250

[B22] Mosborg DA Crane RT Tami TA Parker GS Burn scar carcinoma of the head and neck Arch Otolaryngol Head Neck Surg 1988 11 1038 1040 10.1001/archotol.1988.01860210104027 3408573

[B23] Smith J Mello LF Nogueira Neto NC Meohas W Pinto LW Campos VA Barcellos MG Fiod NJ Rezende JF Cabral CE Malignancy in chronic ulcers and scars of the leg (Marjolin’s ulcer): a study of 21 patients Skeletal Radiol 2001 11 331 337 10.1007/s002560100355 11465774

[B24] Ozek C Celik N Bilkay U Akalin T Erdem O Cagdas A Marjolin’s ulcer of the scalp: report of 5 cases and review of the literature J Burn Care Rehabil 2001 11 1 65 69 10.1097/00004630-200101000-00013 11227688

[B25] Enoch S Miller DR Price PE Harding KG Early diagnosis is vital in the management of squamous cell carcinomas associated with chronic non healing ulcers: a case series and review of the literature Int Wound J 2004 11 165 175 10.1111/j.1742-4801.2004.00056.x 16722875 PMC7951595

[B26] Bauer T David T Rimareix F Lortat-Jacob A Marjolin’s ulcer in chronic osteomyelitis: seven cases and a review of the literature Rev Chir Orthop Reparatrice Appar Mot 2007 11 63 71 17389826 10.1016/s0035-1040(07)90205-6

[B27] Papadopoulos O Frantzoglou M Chrisostomidis C Konofaos P Frangoulis M Barlas G Neglected squamous cell carcinoma of the frontal area: a clinical report J Craniofac Surg 2006 11 1015 1020 10.1097/01.scs.0000234983.61745.38 17003637

[B28] Copcu E Marjolin’s ulcer a preventable complication of bums Plast Reconstr Surg 2009 11 156e 164e 10.1097/PRS.0b013e3181a8082e 19568055

[B29] Emsen IM A great Marjolin’s ulcer of the scalp invading outer calvarial bone and its different treatment with support of medpor J Craniofac Surg 2008 11 1026 1029 10.1097/SCS.0b013e31809eda0d 18650726

[B30] Ko Y Han YM Hwang HS Lee ES Lee GK Role of 18 F-FDG PET/CT in the diagnosis of clinically suspected Marjolin ulcer AJR Am J Roentgenol 2012 11 6 1375 1379 10.2214/AJR.11.8398 23169733

[B31] Hansen SL Mathes SJ Mathes SJ Problem wounds and principles of closure Plastic Surgery 2006 Philadelphia: Saunders Elsevier 916

[B32] Tiftikcioglu YO Ozek C Bilkay U Uckan A Akin Y Marjolin ulcers arising on extremities Ann Plast Surg 2010 11 3 318 320 10.1097/SAP.0b013e3181a73064 20179482

